# Supplementary material for: Flattop regulates basal body docking and positioning in mono- and multiciliated cells
Source: eLife. 2014 Oct 8;3:e03842. doi: 10.7554/eLife.03842 (PMC4221739; doi:10.7554/eLife.03842)
Supplement: Figure 1—source data 1. — (A–C) Fltp animals are born roughly at the expected Mendelian ratio in C57Bl6/6NCrl, 129S6/SvEvTac, or CD1 background. Note: FltpZV/ZV animals are slightly underrepresented on the C57Bl6 and 129S6 background. DOI: http://dx.doi.org/10.7554/eLife.03842.004 [file elife03842s001.pdf]

Gegg et al. Figure 1-source data 1
Mendelian ratio of Fltp intercrosses on different backgrounds

A

Fltp on C57Bl6/6NCrl background G5-6				
	Fltp $^{+/+}$	*Fltp* $^{ZV/ZV}$	*Fltp* $^{ZV/+}$	
total	162	121	279	562
percentage	28.8%	21.5%	49.6%	100%

B

Fltp on 129S6/SvEvTac background G3-4				
	Fltp $^{+/+}$	*Fltp* $^{ZV/ZV}$	*Fltp* $^{ZV/+}$	
total	113	74	176	363
percentage	31.1%	20.4%	48.5%	100%

C

Fltp on CD1 background G2-3				
	Fltp $^{+/+}$	*Fltp* $^{ZV/ZV}$	*Fltp* $^{ZV/+}$	
total	49	45	81	175
percentage	28.0%	25.7%	46.3%	100%
